# Optogenetic Activation of Accessory Olfactory Bulb Input to the Forebrain Differentially Modulates Investigation of Opposite versus Same-Sex Urinary Chemosignals and Stimulates Mating in Male Mice

**DOI:** 10.1523/ENEURO.0010-17.2017

**Published:** 2017-03-23

**Authors:** Tenzin Kunkhyen, Elizabeth A. McCarthy, Wayne J. Korzan, Danielle Doctor, Xue Han, Michael J. Baum, James A. Cherry

**Affiliations:** 1 Department of Psychological and Brain Sciences, Boston University, Boston, MA 02215; 2 Department of Biology, Boston University, Boston, MA 02215; 3 Department of Biomedical Engineering, Boston University, Boston, MA 02215

**Keywords:** olfactory, optogenetic, reproduction, vomeronasal

## Abstract

Surgical or genetic disruption of vomeronasal organ (VNO)-accessory olfactory bulb (AOB) function previously eliminated the ability of male mice to processes pheromones that elicit territorial behavior and aggression. By contrast, neither disruption significantly affected mating behaviors, although VNO lesions reduced males’ investigation of nonvolatile female pheromones. We explored the contribution of VNO-AOB pheromonal processing to male courtship using optogenetic activation of AOB projections to the forebrain. Protocadherin-Cre male transgenic mice received bilateral AOB infections with channelrhodopsin2 (ChR2) viral vectors, and an optical fiber was implanted above the AOB. In olfactory choice tests, males preferred estrous female urine (EFU) over water; however, this preference was eliminated when diluted (5%) EFU was substituted for 100% EFU. Optogenetic AOB activation concurrent with nasal contact significantly augmented males’ investigation compared to 5% EFU alone. Conversely, concurrent optogenetic AOB activation significantly reduced males’ nasal investigation of diluted urine from gonadally intact males (5% IMU) compared to 5% IMU alone. These divergent effects of AOB optogenetic activation were lost when males were prevented from making direct nasal contact. Optogenetic AOB stimulation also failed to augment males’ nasal investigation of deionized water or of food odors. Finally, during mating tests, optogenetic AOB stimulation delivered for 30 s when the male was in physical contact with an estrous female significantly facilitated the occurrence of penile intromission. Our results suggest that VNO-AOB signaling differentially modifies males’ motivation to seek out female vs male urinary pheromones while augmenting males’ sexual arousal leading to intromission and improved reproductive performance.

## Significance Statement

The vomeronasal organ (VNO)-accessory olfactory bulb (AOB) pathway forms a pheromone processing circuit that controls social behaviors of mice. Here, optogenetic activation of AOB forebrain projections increased males’ nasal investigation of diluted (5%) estrous female urine (EFU) but reduced males’ investigation of diluted (5%) testes-intact male urine (IMU). No effects of AOB optogenetic activation were seen when paired with nasal access to either water alone or diluted food odors, or when urinary volatiles alone were present. AOB optogenetic activation also augmented males’ capacity for penile intromission with an estrous female. These findings extend prior studies suggesting that the accessory olfactory system (AOS) motivates male mice to seek out opposite-sex and avoid same-sex pheromones while augmenting sexual arousal and resultant reproductive performance.

## Introduction

Vertebrates respond to chemicals in the environment primarily by means of two anatomically distinct circuits: the main olfactory system (MOS) and the accessory olfactory system (AOS). In the MOS, sensory neurons in the olfactory epithelium of the nasal cavity send axonal projections to the main olfactory bulbs (MOBs) in the forebrain. Sensory neurons of the AOS are found in the vomeronasal organ (VNO) located along the ventral midline of the nasal cavity and send projections to the accessory olfactory bulbs (AOBs) on the dorsal surface of each MOB. The MOS responds to volatile odorants that provide animals (including humans) with information about the chemical environment, whereas the AOS is activated by nonvolatiles accessed during physical contact. Although research has revealed much about the anatomy, receptors, and transductory mechanisms that operate in the VNO, the functional significance of the AOS has remained elusive. While there is strong evidence that the VNO processes chemosignals involved in defensive behavior and aggression (Chamero et al., 2007), the specific role of the VNO in processing cues involved in courtship and reproductive behaviors is less clear. Complete removal of the VNO in male mice does not produce measurable deficits in mating performance (Pankevich et al., 2004); however, it has been heretofore difficult to test the significance of AOS activation once males are engaged in mating with females.

Most studies of AOS function have relied on removal or genetic inactivation of the VNO or AOB to infer function based on observed deficits. An alternative approach using activation of the AOB in awake, behaving subjects has yet to be described. Optogenetic methods in transgenic animals offer the possibility of precisely timed stimulation of AOB neurons while subjects engage in olfactory-dependent behaviors. Such methods have been applied to manipulate activity in the MOB of behaving subjects (Smear et al., 2013; Li et al., 2014; Kermen et al., 2016). These studies indicate that optogenetic activation of the MOB yields a signal that can be detected and acted on by awake behaving animals, suggesting that an optogenetic approach may be a useful way to study the dynamics of chemosignal processing in the olfactory system.

The goal of the present study was to determine whether optogenetic stimulation of subpopulations of AOB projection neurons (mitral and tufted cells, M/T) could affect the responses of male mice to socially relevant chemosignals (referred to here as pheromones) in isolation or in a social context. In particular, we asked whether laser-induced stimulation of AOB M/T cells could accentuate the responses to diluted same- or opposite-sex urinary cues, or enhance aspects of male courtship and mating behaviors observed during interactions with sexually receptive females. To accomplish this, we introduced channelrhodopsin2 (ChR2) into the AOB of Cre-driver mice, in which olfactory expression of Cre is limited to M/T cells in the MOB and AOB. ChR2-expressing cells were activated by blue laser delivered by a chronically implanted optical fiber, which was controlled during odor choice tests by nose poking at designated odor ports, or by experimenter-delivered stimulation during mating tests with sexually receptive females. Our results indicate that optogenetic stimulation of AOB M/T cells both augmented males’ investigation of diluted estrous female urine (EFU) and reduced the investigation of diluted male urine, but only if subjects were allowed nasal access to the odor stimulus. Further, laser activation timed to occur during male investigation of an estrous female increased male mating efficiency, suggesting a potential benefit to ongoing chemosignal sampling by the AOS during courtship.

## Materials and Methods

### Animals

Transgenic mice in which Cre recombinase is driven from a 10-kb fragment of the protocadherin 21 promoter exhibit selective Cre expression in AOB and MOB M/T cells (Nagai et al., 2005). A breeding population of these mice (Pcdh21-Cre) was established in our laboratory from heterozygous Pcdh21-Cre males that were derived from founders obtained from the Mutant Mouse Regional Resource Center (B6.FVB(Cg)-Tg(Cdhr1-cre)KG66Gsat/Mmucd, ID No.036056-UCD), generously provided by Dr. Ian Davison. Heterozygous Pcdh21-Cre males were bred with C57Bl/6 females (obtained from Charles River Laboratories) in breeding rooms kept on a 12/12 h light/dark cycle. Litters were weaned into same-sex groups at three weeks of age and males were genotyped by PCR of tail DNA with Cre-specific primers before transferring to reverse lighting conditions (12/12 h light/dark, lights off at 9:00 A.M.) for the remainder of the study. Heterozygous, Cre-positive males were identified and given sexual experience with behaviorally receptive stimulus females one week after fiber optic implantation. All other animals used in the study were adult C57Bl/6 mice purchased from Charles River Laboratories. All males used were gonadally intact. Stimulus females were ovariectomized on arrival and, to create a condition of behavioral estrus, given subcutaneous injections of 20 µg of estradiol benzoate 48 and 24 h, and 500 µg progesterone 4 h before using in mating behavior tests, as urine donors, and to give sexual experience to male subjects. For sexual experience, male subjects were singly housed and a female was placed in the male’s cage overnight; each male received two such pairings. After the first sexual experience pairing, all male mice were singly housed for the remainder of the experiment. For urine donation, four to six hormonally primed females were placed in metabolic chambers and urine (EFU) was collected for ∼4 h. Urine was collected and stored in 1-ml vials at -70°C until use. Urine from gonadally intact C57Bl/6 males (IMU) was collected similarly. All procedures were approved by the Boston University Charles River Campus Institutional Animal Care and Use Committee.

### Viral infection and fiber optic implantation

Mice were anesthetized with a 2.0% isoflurane gas/oxygen mixture and the head was secured in a sterotaxic apparatus (David Kopf Instruments). The skull was exposed over the olfactory bulbs and small holes were drilled bilaterally to expose the dura. Pulled glass micropipettes (Wiretrol; Drummond Scientific) were filled with an adeno-associated viral vector [AAV5/EF1a-DIO-hChR2(H134)-mCherry-WPRE-pA] created by Karl Deisseroth and distributed by the University of North Carolina Vector Core. This virus (referred to as AAV-ChR2) enables expression of both ChR2 and a fluorescent reporter, mCherry, in cells that express Cre recombinase. Micropipettes were lowered into the brain at a 40° angle (to avoid damaging blood vessels; Kang et al., 2011) at the following coordinates: anterior-posterior, 1 mm rostral to the inferior cerebral vein; medial-lateral, 0.8 mm from the midline; depth, 1.8 mm below the dura. Injections were conducted with a Quintessential Stereotaxic Injector (Stoelting) at the rate of 0.150 µl/min for a total of 0.3 µl. The micropipettes were left in place for an additional 10 min and then slowly withdrawn over the course of 60 s. For chronic implantation of the optical fiber, a small hole was drilled along the midline ∼3.5 mm rostral to bregma, and a single optical fiber (length = 2.0 mm, 200 µm core, numerical aperture = 0.37; Doric Lenses) capped with a stainless steel or zirconia ferrule was stereotaxically lowered into position. Any bleeding from puncturing the inferior cerebral vein was controlled by pressure, after which the ferrule was secured to the skull with the help of two anchoring screws and dental cement. Only one optical fiber was implanted, as the single fiber placed at the midline was sufficient in pilot experiments to drive activity of neurons in the medial amygdala bilaterally (see Fig. 2), which are primary targets of AOB M/T cells. After the dental cement was dry, the incision was closed with suture and mice were given Carprofen (5 mg/kg) analgesic for 2 days and allowed to recover before returning to their home cages. After approximately one week, males were individually housed and given sexual experience as described above. Experiments began three weeks after surgery to insure completeness of viral infection.

### *In vivo* electrophysiological recordings

To demonstrate that virally infected AOB M/T cells produce functional responses upon optical activation, extracellular recordings were conducted in several additional Pcdh21-Cre^+^ males that had been infected with AAV-ChR2 into the AOB at least three weeks earlier. Mice were anesthetized with urethane (10 mg/kg) and placed in a stereotaxic apparatus (David Kopf Instruments) with heating pad to maintain core body temperature. A small hole was drilled over the inferior cerebral vein to allow an optical fiber to be stereotaxically lowered 1.8 mm below the dura along the midline. A second hole was drilled using coordinates described by DiBenedictis et al. (2014) for locating the medial amygdala using the interaural line as a reference point (AP, +2.7 mm; ML, ±2.00 mm; DV, -5.5 mm). A final hole drilled on the ipsilateral side was used to install a reference electrode. A tungsten microelectrode (FHC) attached to a micro-drive was lowered into the medial amygdala. Once spontaneous activity was detected, laser stimulation of the AOB (5-ms pulses, 20 Hz) was applied for 5 s at varying levels of power, and neuronal activity was captured using an amplifier (FHC) and a Micro1401 (Cambridge Electronic Design) data acquisition system. After completion of recordings an electrolytic lesion was made at the site (200 µA, 25 s) to verify placement of the electrode.

### Test boxes and laser delivery

Behavioral tests were conducted in Plexiglas test boxes (26.5 Length × 20 Width × 30 Height cm). For the olfactory choice tests, there were two odor ports on one side 17 cm apart. Odors were used at a volume of 20 µl pipetted onto filter paper positioned ∼1 cm behind the odor ports. Ports were designed such that mice could nasally contact the filter paper, allowing access to both volatile + nonvolatile components of odors. However, one group of mice (see below) received trials in which nasal contact was prevented by placing a wire mesh in front of the filter paper such that only volatiles could be detected. Nose-poke frequency and duration were detected using infrared photo beams at the port entrance controlled by an Arduino UNO board and software (http://arduino.cc). The Arduino board was also used in selected trials to control laser stimulation of the AOB via the chronically implanted optical fiber. At the beginning of each behavioral test, a flexible 1.5 m fiber optic patch cord was gently attached to the ferrule on the animal’s head. Subjects were able to move freely in the box with the attached patch cord, which was directed toward the ceiling where it connected via a rotary joint (Doric Lenses) to a 473 nm laser (Micron Lux). Laser output was controlled by a function generator (BK Precision 4011A), and power was adjusted with the aid of an optical power meter (Thorlabs). Five-millisecond laser pulses were generated at 20 Hz to mimic the maximal firing rate observed in AOB neurons during active nasal investigation of a conspecific (Luo et al., 2003). The laser activated in the selected odor choice tests when a nose-poke was detected and remained on for the duration of the nose-poke.

### Behavioral tests

#### Odor investigation

Male mice were tested in a series of 10-min trials given on separate days in which one port contained an odor and the other port contained the vehicle, and the level of investigation of odors within the odor ports as determined by nose-poking was recorded. At the beginning of each trial, the fiber optic patch cord was gently attached to the implanted ferrule and the subject was placed in the test box for 20 min to habituate to the environment. During this time, the odor ports were blocked to prevent access. After habituation, the ports were unblocked and investigation in each port was recorded for 10 min. Ports were baited with different odors in a vehicle of 20-µl deionized water (urine trials) or mineral oil (food trials) pipetted onto filter paper. Before beginning the odor choice series of tests, mice in all groups were given four habituation trials on four successive days to familiarize subjects to the test box and to laser stimulation. The goal of these trials was twofold: to pre-expose subjects to the experience of optical activation before odor choice trials were initiated, and to insure that optogenetic activation of AOB M/T cells, in the absence of odors, was not inherently rewarding or aversive. In these trials, both odor ports were baited with deionized water and one port was randomly chosen on the first trial to produce laser stimulation on nose-poking. The next three trials were identical except that the port coupled with laser stimulation was alternated each day. After completion of these trials, olfactory choice tests were given to assess whether laser activation of the AOB could affect investigation of a suboptimal stimulus (diluted urine). In pilot studies, male mice consistently showed no preference for investigating a 5% dilution of EFU compared with water (data not shown), so this dilution was used for both EFU and IMU in trials where diluted urine preferences were examined.

Three separate groups of mice received the olfactory choice tests (Table 1). Specifically, group 1 subjects (*n* = 9) were Pcdh21-Cre-positive and had received bilateral AOB injections of AAV-ChR2. These mice received three series of trials in which nasal access to both volatile and nonvolatile components of the odor was allowed. One series consisted of five trials with EFU used as the odor source. In trial 1, 100% EFU was placed behind one port and water behind the second port; trial 2 was identical to trial 1 except that the ports with EFU and water were switched. On trial 3, one port was baited with 5% EFU and investigation at this port also produced laser stimulation for the duration of the nose poke. On trial 4, 5% EFU without laser stimulation was used, and trial 5 was a replication of trial 3. A second series of trials was given in the same manner as the first series, except that IMU was used in place of EFU in one of the odor ports. A sixth trial was also added that repeated the trial 4 test of 5% IMU alone to insure that repeated testing had not produced lack of interest in investigation of the odor ports. For the final series of odor choice trials a food odor was used in place of urine. The food was a paste of NutterButter cookie (Nabisco) in mineral oil; mineral oil was used in the other odor port. Several days before the odor choice tests, subjects had been fed the cookies, which they eagerly consumed. Six trials were given as was done with the IMU test series, with 5% food odor being paired with laser on trials 3 and 5; 100% food odor was used in trial 6. To insure sufficient investigation of cookie odors during testing, mice were food restricted (2-h access to food pellets each day) while these trials were being conducted. Group 2 (*n* = 8) subjects were Pcdh21-Cre-positive and had received bilateral AOB injections of AAV-ChR2. These mice were tested in the same series of trials with EFU, IMU, and food as the odor stimulus that the group 1 subjects received, except that only access to volatiles was permitted. In addition, after the olfactory choice tests, these subjects were given habituation-dishabituation tests (e.g., Baum and Keverne, 2002) to insure that the ability to discriminate between diluted urinary odors was intact. Briefly, these subjects were given three presentations of water followed by three presentations of 5% IMU followed by three presentations of 5% EFU. Subjects received these tests in their home cage and had nasal access to the odor cues applied to filter paper. The time that mice spent investigating (nose within 1 cm) the odor was recorded. After the habituation-dishabituation testing, these subjects were used in mating tests to examine the effects of laser stimulation on sexual behavior (described below). Finally, to control for the possibility that laser stimulation by itself had an effect on the investigation of urine, a third group (group 3, C57Bl/6 males, *n* = 7) of subjects was tested that did not receive an injection of AAV-ChR2 but were fitted with the chronic fiber optic implant and received laser stimulation during selected trials with diluted urine. These mice received the EFU and IMU series of trials in which access to both volatile and nonvolatiles was permitted.

**Table 1.** Summary of experimental details and AAV-ChR2 infection rates for male subjects

Group	*n*	Genotype	AOB AAV-ChR2 infection	Access to olfactory stimuli	Olfactory preference tests	Mating tests	Average number of infected cells per section	
							L	R
1	9	Pcd21-Cre^+^	Y	Volatiles + nonvolatiles	EFU vs water;	N	11.1±0.74	9.44±0.8
					IMU vs water;			
					food vs water			
2	8*	Pcd21-Cre^+^	Y	Volatiles only	EFU vs water;	Y	13.4±1.41	12±1.21
					IMU vs water;			
					food vs water			
3	7	C57BL/6	N	Volatiles + nonvolatiles	EFU vs water;	N	-	-
					IMU vs water			

*Only seven males were used in the mating tests.

#### Mating behavior

After completing olfactory choice and chemosignal habituation-dishabituation testing, group 2 mice (Pcdh21-Cre-positive and infected with AAV-ChR2) were observed in mating tests with estrous females. Male subjects (one group 2 male could not be used, leaving *n* = 7) were connected to the fiber optic cable and, to facilitate habituation, placed into a test box containing ∼500-ml 50% clean bedding and 50% soiled bedding from the male’s own cage to make the odor environment more similar to the male's home cage. After 30 min of habituation, a sexually receptive female was introduced into the test box and mating behaviors were observed. Each male subject received three tests, conducted four days apart and in randomized order. For test 1 (baseline), males received no laser stimulation during the test. For test 2 (timed), the laser was turned on for 30 s every 3 min, regardless of males’ location or behavior toward the stimulus female. [Thirty-second laser stimulation was chosen based on Luo et al. (2003), who showed that in freely moving mice, AOB mitral cell firing persisted for up to 30 s after initiating nasal investigation of an anesthetized stimulus animal.] For test 3 (triggered), optogenetic AOB stimulation was delivered for 30 s when the male subject either engaged in close nasal investigation of the female for at least 1 s, or mounted the female. After the 30-s laser stimulation, no laser activation occurred for 2.5 min. After the 2.5-min interval, the next investigation or mount of the female by the male produced another delivery of 30-s laser stimulation, followed again by 2.5 min of no activation. This pattern continued until the test was terminated, which occurred after males ejaculated or after 20 min had elapsed. Therefore, each timed test that lasted 20 min contained 3 min (6 × 30 s) during which the laser was on; full-length triggered tests could contain as much as 3 min of laser stimulation but could contain less if males failed to investigate the female. Tests were videotaped and subsequently viewed to score males’ investigation of the female (defined as nasal contact with the body of the estrous female) as well as mounting of the female. Mounts with slow rhythmic pelvic thrusting were distinguished and scored as intromissions (Baum et al., 1994; Morali et al., 2003).

### Histologic assessment of viral infection

Once the behavioral assays were completed, all animals were deeply anesthetized with sodium pentobarbital (150 mg/kg i.p.) and were transcardially perfused with 0.1 M PBS, pH 7.4, and then with 4% paraformaldehyde (PFA). Brains were extracted from the skull and postfixed in 4% PFA for 2 h and then cryoprotected in 30% sucrose for 48 h at 4°C. Olfactory bulbs and cortex tissue were stored in embedding compound (Tissue-Tek OCT, Sakura) at -80°C until sectioning. Tissue was cut in 30-µm sagittal sections using a cryostat (Microm HM 500M, Richard Allen Scientific). Sections were mounted on slides with Vectashield mounting medium, in some cases with 4’,6-diamidino-2-phenylindole (DAPI; 1.5 μg/ml; Vector Laboratories) as a counterstain. Microscopy images were acquired using a Nikon Ni-E fluorescent motorized microscope or an Olympus Fluoview FV10i inverted confocal laser scanning microscope. Three representative sections (medial, middle, and lateral, ∼150 μm apart) were examined for each AOB hemisphere, which was identified based on its dorsal position and unique laminar structure. The total number of red fluorescent M/T cells (i.e., mCherry+) were counted using ImageJ Software (NIH). To obtain a rough estimate of the proportion of M/T cells that were infected, all DAPI-labeled neurons in the mitral cell layer of these sections were counted for two subjects that received viral injections to estimate the total number of cells per AOB in each hemisphere (four hemispheres). These estimates were then used to compute the percentage of M/T cells that were infected with ChR2-mCherry in each hemisphere.

### Statistical analysis

Data from the laser pre-exposure trials with water in each odor port, as well as the odor choice tests were examined for each group of subjects using two-way repeated measures ANOVAs with trial and odor port as the factors. Analyses were conducted using the General Linear Models procedure in IBM SPSS Statistics; data from each series of trials with different odors (pre-exposure, EFU, IMU, or food) were analyzed separately. Huynh-Feldt corrections were applied when the sphericity assumption of repeated measures ANOVAs was violated; the corrected degrees of freedom are reported where appropriate in Table 2. Planned (a priori) comparisons were used to examine whether laser stimulation coupled with odors differed from investigation of odor alone. Other planned comparisons examined investigation of 100% vs 5% odor (no laser), or 100% odor versus 5% odor + laser. Habituation-dishabituation was examined with paired *t* tests to compare the mean investigation times for the dishabituation responses of each group: third water presentation versus the first EFU, and the third presentation of EFU with the first presentation of IMU. In the mating tests, investigation rates and measures of sexual behavior were compared between the baseline, timed, and triggered tests with repeated measures ANOVAs. Because the first laser stimulus was not applied in the timed and triggered tests until at least 2.5 min into the test, behaviors occurring in the first 2.5 min of all tests were not included in analyses. In the two tests with laser activation, paired *t* tests were used within each test to compare behaviors occurring with the laser on versus those occurring when the laser was off.

**Table 2:** Summary of statistical analyses for data shown in Figures 3–7

	Type of test*	Analysis	Test value#	*p* value	Observed power
a	Two-way RM ANOVA	Main effect, odor port	*F*_(1,8)_ = 0.03	0.88	0.052
b	Two-way RM ANOVA	Main effect, odor port	*F*_(1,7)_ = 4.23	0.08	0.427
c	Two-way RM ANOVA	Main effect, odor port	*F*_(1,6)_ = 3.59	0.11	0.358
d	Two-way RM ANOVA	Main effect, odor port	*F*_(1,8)_ = 15.1	0.005	0.923
e	Two-way RM ANOVA	Main effect, trial	*F*_(1.65,15.7)_ = 7.96	0.007	0.861
f	Two-way RM ANOVA	Interaction, odor port × trial	*F*_(1.97,15.7)_ = 6.56	0.009	0.841
g	Two-way RM ANOVA	Planned comparison, trials 3 and 5 vs trial 4	*F*_(1,8)_ = 15.2	0.005	0.925
h	Two-way RM ANOVA	Planned comparison, trials 1 and 2 vs trial 4	*F*_(1,8)_ = 12.0	0.009	0.856
i	Two-way RM ANOVA	Planned comparison, trials 1 and 2 vs trials 3 and 5	*F*_(1,8)_ = 7.46	0.026	0.668
j	Two-way RM ANOVA	Main effect, odor port	*F*_(1,8)_ = 1.79	0.218	0.219
k	Two-way RM ANOVA	Main effect, trial	*F*_(2.8,22.7)_ = 2.50	0.088	0.528
l	Two-way RM ANOVA	Interaction, odor port × trial	*F*_(5,40)_ = 4.08	0.004	0.924
m	Two-way RM ANOVA	Planned comparison, trials 1 and 2 vs trials 4 and 6	*F*_(1,8)_ = 0.14	0.722	0.062
n	Two-way RM ANOVA	Planned comparison, trials 3 and 5 vs trials 4 and 6	*F*_(1,8)_ = 10.3	0.012	0.802
o	Two-way RM ANOVA	Planned comparison, trials 3 and 5 vs trials 1 and 2	*F*_(1,8)_ = 17.6	0.003	0.955
p	Two-way RM ANOVA	Main effect, odor port	*F*_(1,8)_ = 205.0	0.0001	1.000
q	Two-way RM ANOVA	Planned comparison, trials 1, 2 and 6 vs trial 4	*F*_(1,8)_ = 63.6	0.0001	1.000
r	Two-way RM ANOVA	Planned comparison, trials 1, 2 and 6 vs trials 3 and 5	*F*_(1,8)_ = 83.1	0.0001	1.000
s	Two-way RM ANOVA	Planned comparison, trials 3 and 5 vs trial 4	*F*_(1,8)_ = 4.85	0.059	0.491
t	Two-way RM ANOVA	Main effect, odor port	*F*_(1,7)_ = 33.3	0.0007	0.998
u	Two-way RM ANOVA	Planned comparison, trials 1 and 2 vs trial 4	*F*_(1,7)_ = 22.4	0.002	0.980
v	Two-way RM ANOVA	Planned comparison, trials 3 and 5 vs trial 4	*F*_(1,7)_ = 2.05	0.195	0.237
w	Two-way RM ANOVA	Main effect, trial	*F*_(5,35)_ = 7.88	0.0001	0.998
x	Two-way RM ANOVA	Main effect, odor port	*F*_(1,7)_ = 0.009	0.925	0.051
y	Two-way RM ANOVA	Interaction, odor port × trial	*F*_(5,35)_ = 1.30	0.285	0.404
z	Two-way RM ANOVA	Planned comparison, trials 1 and 2 vs trials 4 and 6	*F*_(1,7)_ = 10.4	0.014	0.790
aa	Two-way RM ANOVA	Planned comparison, trials 1 and 2 vs trials 3 and 5	*F*_(1,7)_ = 10.3	0.015	0.785
bb	Two-way RM ANOVA	Planned comparison, trials 3 and 5 vs trials 4 and 6	*F*_(1,7)_ = 0.026	0.876	0.052
cc	Two-way RM ANOVA	Main effect, odor port	*F*_(1,7)_ = 53.6	0.0002	1.000
dd	Two-way RM ANOVA	Planned comparison, trials 1, 2 and 6 vs trial 4	*F*_(1,7)_ = 47.4	0.0002	1.000
ee	Two-way RM ANOVA	Planned comparison, trials 1, 2 and 6 vs trials 3 and 5	*F*_(1,7)_ = 36.4	0.001	0.999
ff	Two-way RM ANOVA	Planned comparison, trials 3 and 5 vs trial 4	*F*_(1,7)_ = 0.136	0.282	0.173
gg	*t* test (paired)	Third water trial vs first IMU trial	*t*_6_ = 2.66	0.04	0.604
hh	*t* test (paired)	Third IMU trial vs first EFU trial	*t*_6_ = 4.12	0.006	0.926
ii	Two-way RM ANOVA	Main effect, odor port	*F*_(1,6)_ = 43.1	0.0006	1.000
jj	Two-way RM ANOVA	Main effect, trial	*F*_(4,24)_ = 10.2	0.0001	0.999
kk	Two-way RM ANOVA	Interaction, odor port × trial	*F*_(4,24)_ = 17.7	0.0001	1.000
ll	Two-way RM ANOVA	Planned comparison, trials 1 and 2 vs trial 4	*F*_(1,6)_ = 17.3	0.006	0.931
mm	Two-way RM ANOVA	Planned comparison, trials 1 and 2 vs trials 3 and 5	*F*_(1,7)_ = 36.2	0.001	0.998
nn	Two-way RM ANOVA	Planned comparison, trials 3 and 5 vs trial 4	*F*_(1,6)_ = 3.45	0.113	0.347
oo	Two-way RM ANOVA	Main effect, odor port	*F*_(1,6)_ = 1.85	0.223	0.210
pp	Two-way RM ANOVA	Main effect, trial	*F*_(2.17,13.0)_ = 2.62	0.107	0.445
qq	Two-way RM ANOVA	Interaction, odor port × trial	*F*_(2.3,14.0)_ = 3.03	0.075	0.526
rr	Two-way RM ANOVA	Planned comparison, trials 1 and 2 vs trials 4 and 6	*F*_(1,6)_ = 2.38	0.174	0.256
ss	Two-way RM ANOVA	Planned comparison, trials 1 and 2 vs trials 3 and 5	*F*_(1,6)_ = 3.76	0.101	0.372
tt	Two-way RM ANOVA	Planned comparison, trials 3 and 5 vs trials 4 and 6	*F*_(1,6)_ = 1.87	0.221	0.212
uu	*t* test (paired)	Timed: intromissions, laser-ON vs laser-OFF	*t*_6_ = 0.389	0.711	0.063
vv	*t* test (paired)	Triggered: intromissions, laser-ON vs laser-OFF	*t*_6_ = 3.27	0.017	0.777

Letters in first column refer to tests shown in the Results. RM, repeated measures.

* All tests based on normal distribution.

# Tests that violated the sphericity assumption are shown after Hunyh-Feldt correction.

## Results

### Anatomic and functional verification of ChR2 expression

**Figure 1.**
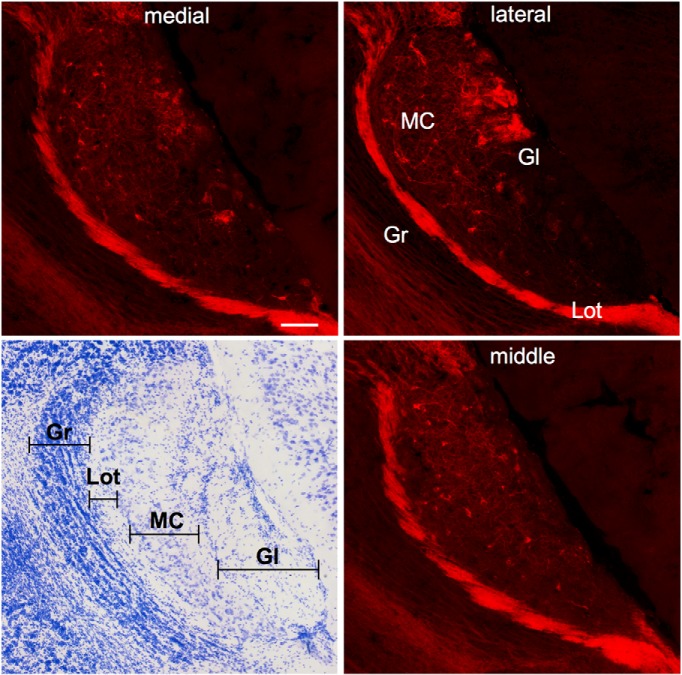
Examples of the distribution of ChR2-mCherry-infected mitral cells in the AOB in three sagittal sections from medial, middle, and lateral regions of a single representative AOB. mCherry-labeled cells and fibers can be seen throughout the mitral cell layer, and fibers are also prevalent in the glomerular layer (Gl) and lateral olfactory tract (Lot). A photomicrograph of a Nissl-stained section from the AOB is shown on the bottom left panel. Gr, granule cell layer; MC, mitral cell layer. Scale bar = 100 microns.

**Figure 2.**
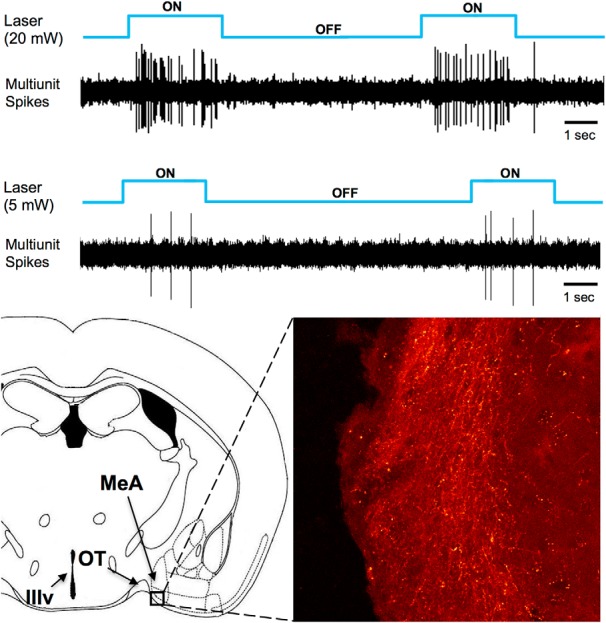
Top, Multiunit activity in the medial amygdala of an anesthetized, Pcdh21-Cre male mouse virally infected with ChR2 in the AOB is shown following optogenetic stimulation of the AOB. Traces are shown following stimulation at laser power levels of 20 and 5 mW. Bottom, ChR2-mCherry fluorescing axons appear coursing through the medial amygdala. MeA, anterior medial amygdala; OT, optic tract; IIIv, third ventricle. The boxed region in the MeA is ∼ 250 × 250 microns

Bilateral infection of the AOB with AAV-ChR2 yielded, as expected, expression of mCherry that was mainly restricted to cells in the mitral cell layer of the AOB (Fig. 1), and red fluorescing fibers were also seen in the anterior medial amygdala (MeA), a primary target of AOB output (Fig. 2). Although we also noted some spread of infection to adjacent M/T cells in the dorsal MOB of most subjects, this labeling was sporadic and distant from the site of the optical fiber. There was an average of between nine and eleven mCherry+ cells per AOB section in male mice that received injections of AAV-ChR2 virus (Table 1). Based on the total number of DAPI-labeled cells counted in four hemispheres, the infection rate for each hemisphere was estimated to be between 2% and 6% of all AOB cells in the mitral cell layer. As DAPI stains glia in addition to neurons (Loesel et al., 2006), these values underestimate the proportion of the M/T cell population that was infected by the AAV-ChR2 virus. Electrical recordings from the medial amygdala in anesthetized mice demonstrated that optical stimulation of the AOB in Pcdh21-Cre-positive mice infected with AAV-ChR2 produced spiking activity (Fig. 2). The lowest power that produced reliable spiking was 5 mW at the tip of the optical fiber; this level was used for all of the behavioral studies.

### Odor investigation

**Figure 3.**
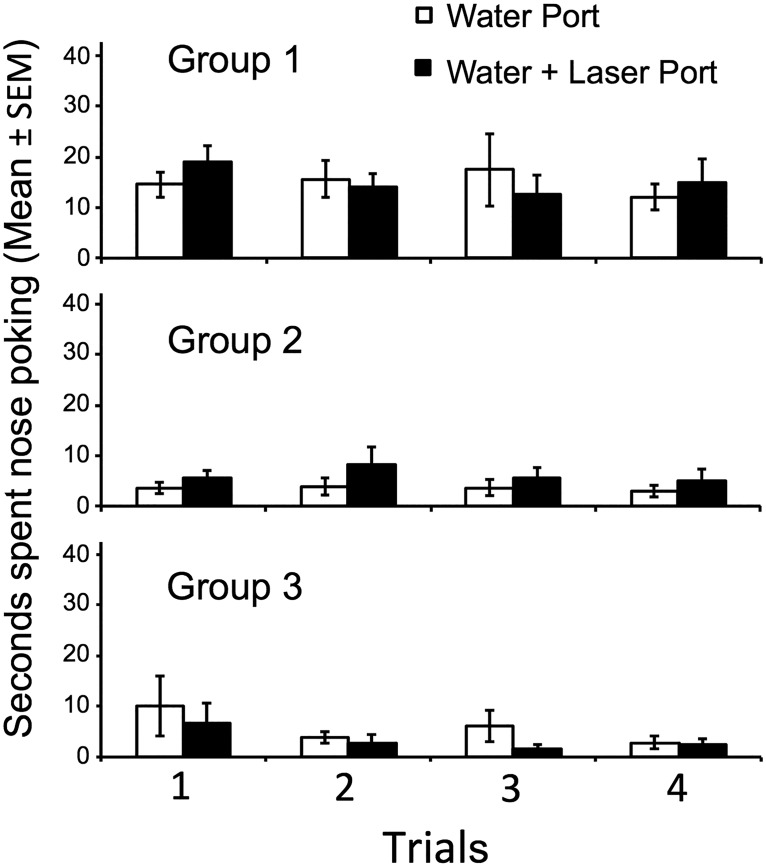
Blue laser stimulation of the AOB when paired with the investigation of water in one test port failed to either augment or reduce the time that male mice spent investigating at that port. ***A***, group 1, Protocadherin-Cre subjects in which AOB mitral cells were infected with ChR2 virus (AAV-ChR2); nasal access to the water stimuli was allowed. ***B***, group 2, Protocadherin-Cre subjects in which AOB mitral cells were infected with AAV-ChR2; nasal access to the water stimuli was blocked. ***C***, group 3, C57Bl/6 subjects without viral infection; nasal access to the water stimuli was allowed.

For pre-exposure trials, water versus water + laser, all groups received four trials with both odor ports baited with water before beginning odor choice trials to determine whether laser activation coupled with one of the odor ports would affect investigation. For none of the three groups was there a main effect of odor port (group 1, *p* = 0.88; group 2, *p* = 0.08; group 3, *p* = 0.11)_abc_, indicating that laser stimulation by itself did not affect investigation (Fig. 3).

#### Group 1: Pcdh-Cre^+/-^, AAV-ChR2, volatiles + nonvolatiles

**Figure 4.**
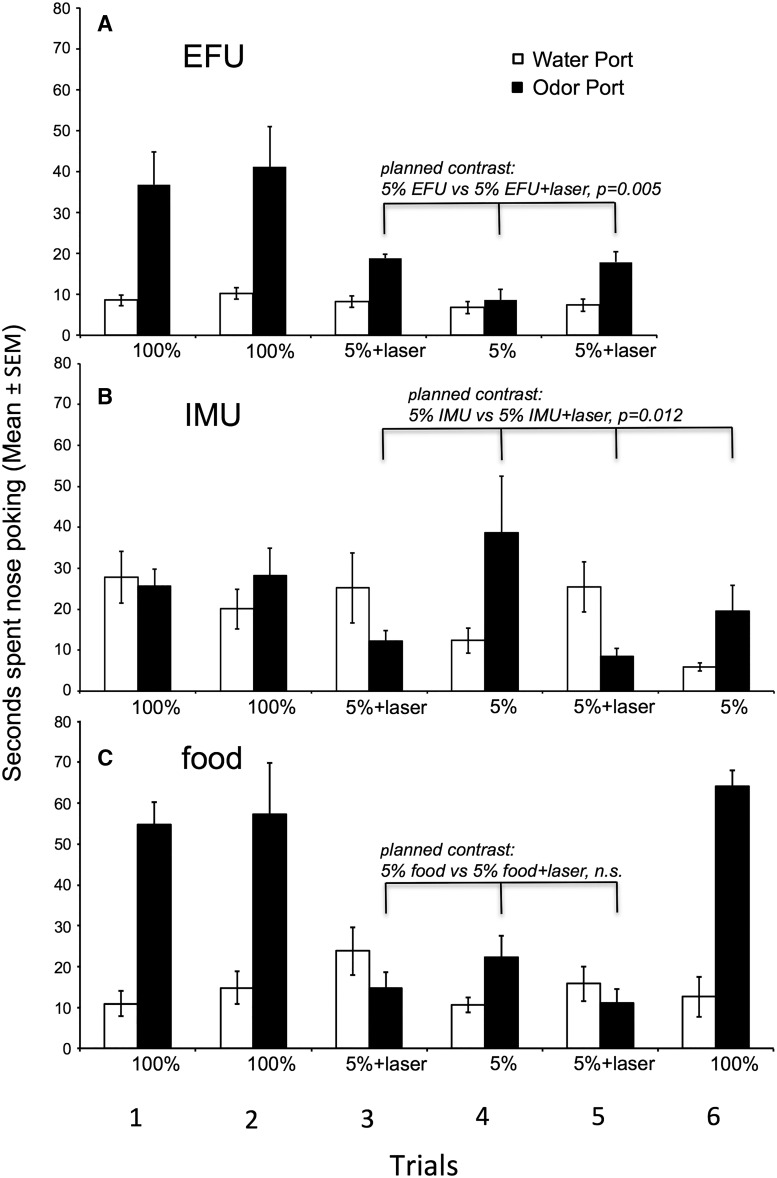
Effect of optogenetic stimulation of the AOB on the preference of male mice (group 1) to investigate (nose poke) ports containing water versus one of three different odors in trials given on separate days. Subjects were permitted nasal access to odors/water in all trials. ***A***, Overall, male subjects preferred to investigate female urinary odors over water (main effect of odor port). A planned contrast revealed that optogenetic AOB stimulation significantly augmented males’ preference for 5% EFU compared with 5% EFU alone. Other planned contrasts (not indicated) showed that males investigated 100% EFU more than either 5% EFU or 5% EFU coupled with laser. ***B***, Overall, male subjects showed no preference to investigate IMU over water (no main effect of odor port). A planned contrast showed that optogenetic AOB stimulation significantly reduced males’ investigation of 5% IMU relative to 5% IMU alone. An additional planned contrast (not indicated) showed that there was no difference in the investigation of 100% IMU versus 5% IMU. ***C***, Male subjects preferred to investigate food odors over water (main effect of odor port). A planned contrast showed that pairing optogenetic AOB stimulation with 5% food odors failed to affect males’ investigation of this stimulus.

In the first series of trials, mice investigated urinary odors from estrous females significantly more than the water port (Fig. 4*A*), as indicated by a main effect for odor port (*p* = 0.005)_d_, and overall investigation of odor ports differed among the five trials (trial effect, *p* = 0.007)_e_. There was also a significant trial × odor port interaction (*p* = 0.009)_f_. Planned comparisons revealed that laser stimulation coupled with 5% EFU (trials 3 and 5) significantly enhanced investigation relative to 5% EFU alone (trial 4, *p* = 0.005)_g_. Investigation of 100% EFU (trials 1 and 2) was significantly greater than either 5% EFU alone (trial 4, *p* = 0.009)_h_ or 5% EFU with laser (trials 3 and 5, *p* = 0.026)_i_. These results indicate that although laser activation coupled with 5% EFU increased investigation relative to 5% EFU alone, it only partially duplicated the effects of 100% EFU on males’ motivation to investigate.

Quite different results were seen in tests with IMU (Fig. 4*B*). Overall, there was no difference in the investigation of 100% (undiluted) IMU stimuli compared with water (odor port effect, *p* = 0.218_j_), and investigation did not differ across the six trials (trial effect, *p* = 0.088)_k_, but there was a trial × odor port interaction (*p* = 0.004)_l_. Planned comparisons revealed that while there was no difference in the mean investigation of 100% IMU compared with 5% IMU (*p* = 0.722)_m_, levels of investigation during laser pairing with 5% IMU were significantly reduced relative to either 5% IMU alone (*p* = 0.012)_n_ or 100% IMU (*p* = 0.003)_o_. These results indicate that laser activation of the AOB coupled with 5% IMU revealed an aversion for this stimulus not seen in male subjects exposed to 100% IMU.

To examine whether the enhanced response to 5% EFU when coupled with laser activation might generalize to a different attractive odor, a food source was used (Fig. 4*C*). Food (NutterButter) odor proved highly attractive to mice relative to water (odor port effect, *p* < 0.0001)_p_. Planned comparisons showed that 100% food odor was preferred over either 5% food odor alone (*p* < 0.0001)_q_ or 5% food odor + laser (*p* < 0.0001)_r_. There was a nonsignificant trend for investigation of 5% food odor + laser to be less than that for 5% food odor alone (*p* = 0.059)_s_. As the food odors presented in this study were presumably detected by the MOS, it is not surprising that optogenetic activation of the AOB had no effect on subjects’ investigation of this stimulus.

#### Group 2: Pcdh-Cre^+/-^, AAV-ChR2, volatiles only

**Figure 5.**
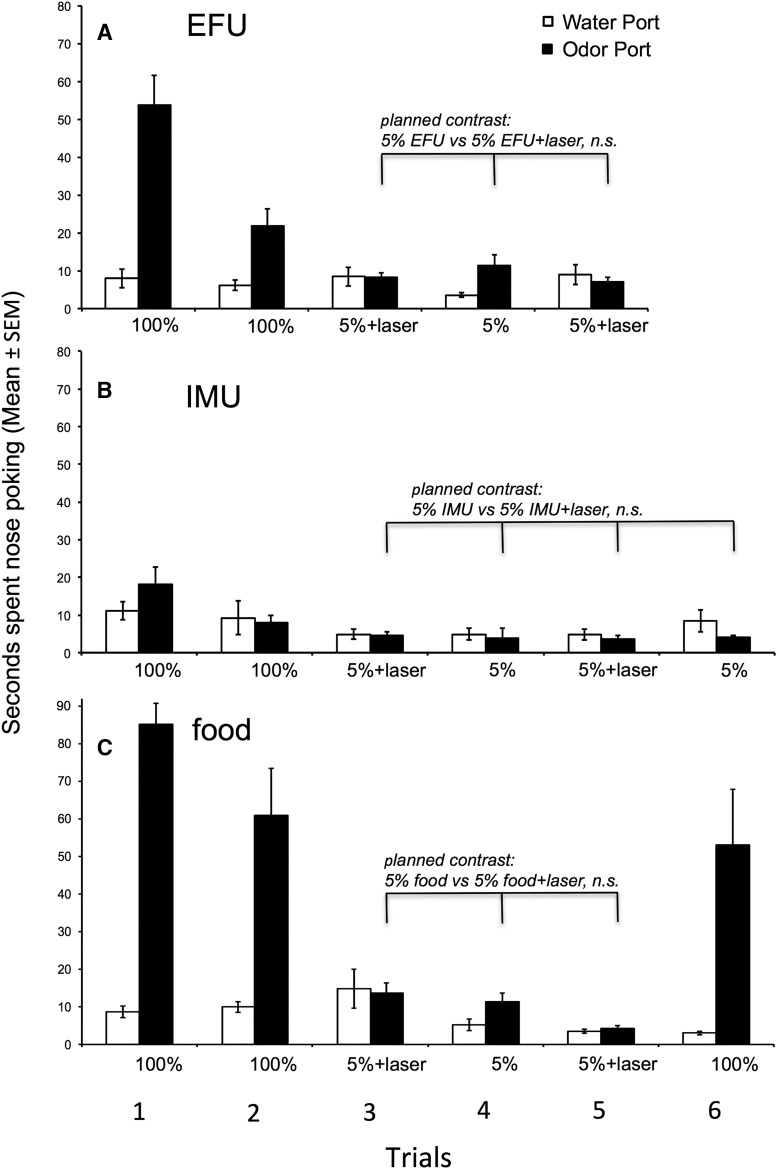
Effect of optogenetic stimulation of the AOB on the preference of male mice (group 2) to investigate (nose poke) ports containing water versus one of three different odors in trials given on separate days. Nasal access to odors/water was blocked, thereby making only volatile odor stimuli available to subjects. ***A***, Male subjects preferred to investigate EFU over water (main effect of odor port). A planned contrast showed that in absence of nasal contact with the stimuli optogenetic AOB stimulation failed to influence males’ preference for 5% EFU over 5% EFU alone. Other planned contrasts (not indicated) showed that investigation of volatiles from 100% EFU was greater in comparison to 5% EFU or 5% EFU plus laser stimulation. ***B***, Male subjects showed no preference to investigate IMU over water (main effect of odor port). In the absence of nasal contact with the stimuli, a planned contrast showed that optogenetic AOB stimulation failed to affect males’ investigation of 5% IMU. ***C***, Male subjects preferred to investigate food odors over water (main effect of odor port). A planned contrast shows that pairing optogenetic AOB stimulation with 5% food odors failed to affect males’ investigation of this stimulus.

To examine whether the increased attractiveness of 5% EFU seen with laser activation in group 1 subjects would be observed when physical contact with the odor stimuli was prevented, a new cohort of subjects (group 2) were tested that were allowed access to volatiles only. As previous research (Martel and Baum, 2007, 2009; Slotnick et al., 2010) suggested that volatile pheromonal signals detected by the MOS gain access to the AOB via centrifugal inputs from the medial amygdala, this experiment tested the possible contribution of such signaling in the context of the stimulatory effects of optogenetic activation of the AOB on males’ investigation of diluted EFU as well as IMU. As was seen for group 1, in trials with EFU versus water male subjects investigated EFU volatiles more than water (odor port effect, *p* = 0.0007)_t_, and 100% EFU was investigated more than 5% EFU (planned comparison of trials 1 and 2 vs trial 4, *p* = 0.002)_u_ (Fig. 5*A*). However, in contrast to group 1, there was no enhancement of investigation of 5% EFU + laser stimulation relative to investigation of 5% EFU alone (planned comparison of trials 3 and 5 vs trial 4, *p* = 0.195)_v_.

In trials with IMU versus water, there was a significant main effect of trial (*p* < 0.0001)_w_, but no effect of odor port (*p* = 0.925)_x_ and no trial × odor port interaction (*p* = 0.285)_y_ (Fig. 5*B*). Planned comparisons found that while 100% IMU was investigated more than either 5% IMU alone (*p* = 0.014)_z_ or 5% IMU + laser (*p* = 0.015)_aa_, there was no difference in the investigation of 5% IMU alone versus 5% IMU + laser (*p* = 0.876)_bb_.

Mice without nasal access to odors responded to food volatiles alone similarly to how they responded to the food stimulus when physical access was allowed. Thus, food odor was highly preferred relative to water (odor port effect, *p* = 0.0002)_cc_, and 100% food odor was preferred over either 5% food odor alone or 5% food odor + laser (planned comparisons, *p* = 0.0002_dd_; *p* = 0.001, respectively)_ee_ (Fig. 5*C*). There was no difference in the investigation of 5% food odor compared with 5% food odor + laser (*p* = 0.282)_ff_.

To insure that the minimal investigation of diluted volatile murine chemosignals was not due to males’ inability to detect the stimuli, subjects were given habituation-dishabituation tests with sequential, home cage exposures to diluted urinary volatiles or water as described previously (Baum and Keverne, 2002). Subjects dishabituated from the final of three presentations of water to the first presentation of 5% IMU (*p* = 0.04)_gg_ as well as from the final of three presentations of 5% IMU to the first of three presentations of 5% EFU (*p* = 0.006)_hh_, indicating that mice were capable of detecting the diluted urinary chemosignals used in the odor preference tests (data not shown).

#### Group 3: C57Bl/6, volatiles + nonvolatiles

**Figure 6.**
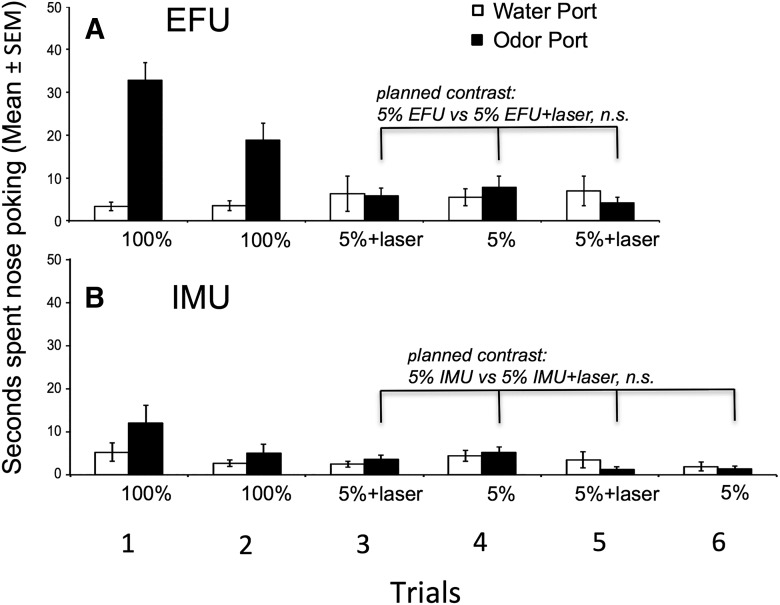
Effects of laser stimulation of the AOB in control (uninfected, nontransgenic) C57Bl/6 male mice (group 3) on the investigation (nose poking) of ports containing water versus urinary odors in trials given on separate days. Nasal access to odors/water was permitted on all trials. ***A***, Male mice preferred to investigate (nose-poke) EFU over water (main effect of odor port); however, a planned contrast found that laser stimulation of the AOB in the absence of ChR2 expression in AOB mitral cells failed to augment males’ investigation of diluted, 5% EFU. ***B***, Male mice never displayed a preference for IMU over water (no main effect of odor port), and a planned contrast showed that laser stimulation of the AOB in the absence of ChR2 expression in AOB mitral cells did not modulate these preferences.

To control for the possibility that laser stimulation alone (in absence of ChR2 expression in AOB M/T neurons) affected investigation of odors, nontransgenic C57Bl/6 males were implanted with a chronic fiber optic and given the series of tests with EFU and IMU. These males all received mating experience with estrous female mice before the urinary preference tests, as well as pre-exposure trials with ports baited with either water or water + laser, similar to groups 1 and 2. For the EFU versus water trials, there were significant main effects of odor port (*p* = 0.0006)_ii_ and trial (*p* < 0.0001)_jj_, as well as a trial × odor port interaction (*p* < 0.0001)_kk_, indicating that odors were investigated differentially in comparison to water across trials (Fig. 6*A*). Planned comparisons showed that 100% EFU was investigated significantly more than either 5% EFU alone (*p* = 0.006)_ll_ or 5% EFU + laser (*p* = 0.001)_mm_, but there was no difference in investigation of 5% EFU alone versus 5% EFU + laser (*p* = 0.113)_nn_. In IMU trials, there was no effect of odor port (*p* = 0.223)_oo_, indicating that overall, water and IMU were investigated equivalently (Fig. 6*B*). Similarly, there was no significant effect of trial (*p* = 0.107)_pp_ and no interaction (*p* = 0.075)_qq_. Further, none of the planned comparisons were significant: 100% IMU versus either 5% IMU alone or 5% IMU + laser, or 5% IMU alone versus 5% IMU + laser (all p > 0.05)_rr-tt_. Together, these results indicate that laser stimulation of the AOB in males not infected with AAV-ChR2 had no effect on response to either EFU or IMU.

### Mating behavior

Males in group 2 were given three mating behavior tests on different days with estrous females, one with the laser off (baseline), one with laser activation occurring at set times during the test (timed), and one with laser activation that was triggered by close investigation of the female by the male subject (triggered). Because tests were terminated if the male ejaculated, test durations varied and investigation rates were computed as percentage of the entire test spent investigating. Notably, in the tests that lasted the full 20 min, all subjects during the triggered test received six 30-s laser activations, which was the same amount as that received in the timed tests.

**Figure 7.**
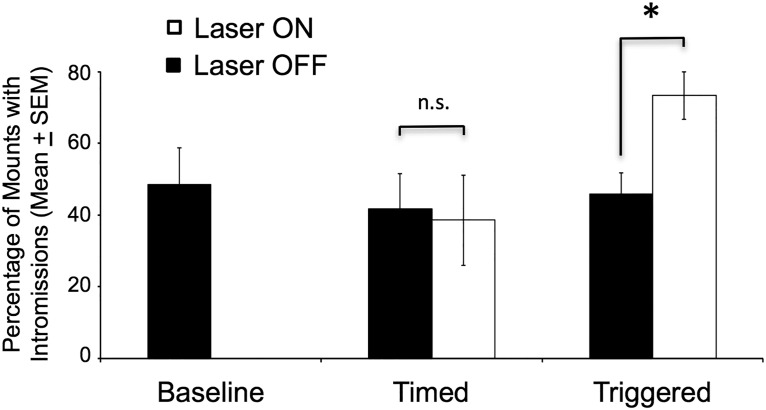
In mating tests, optogenetic stimulation of AOB mitral cells significantly augmented the occurrence of penile intromission in male mice when the laser was switched on following nasal contact the stimulus estrous female or a direct mount of the female (triggered). * denotes a significant difference in the percentage of mounts with intromission during the laser-ON versus laser-OFF portions of the triggered test (paired *t* test, *p* = 0.017). By contrast, there was no difference (n.s.) in males’ intromission success during laser-ON compared with laser-OFF periods when optogenetic AOB stimulation was applied at arbitrary (2.5 min) intervals (timed). Results are also shown from a single baseline test during which the male subjects were allowed to mate with an estrous female in the absence of any optogenetic AOB stimulation.

Two of seven, one of seven, and three of seven males ejaculated in the baseline, timed, and triggered laser tests, respectively; these proportions did not differ significantly between groups. There were also no differences between the three tests in test duration or in various measures of male copulatory behavior, including total number of mounts or intromissions, or in assessment of mating efficiency, which can be defined as the percentage of mounts that include intromissions (“hit rate”; Sachs and Barfield, 1976; Table 3). However, when the timed and triggered tests were divided into laser-ON versus laser-OFF periods, significant effects of laser activation on male mounting behaviors were seen. Specifically, although mating efficiency during the laser-ON versus laser-OFF portions of the test did not differ in the timed tests (*p* = 0.711)_uu_, this measure was significantly higher in the triggered tests during laser-ON compared with laser-OFF periods (*p* = 0.017)_vv_, indicating that only properly timed laser stimulation yielded an increase in mating efficiency (Fig. 7).

**Table 3:** Additional data from mating tests

	Baseline		Timed		Triggered				
Measure	Mean	SEM	Mean	SEM	Mean	SEM	Test value†	*p* value	Observed power
Test duration (s)*	1005.7	*133.9*	1137.6	*62.43*	1048.9	*108.9*	F_1.1,6.4_=0.40	0.564	0.085
Total number of mounts	37.3	*7.09*	36.7	*7.98*	36.3	*5.95*	*F*_(2,12)_ = 0.01	0.992	0.051
Total number of intromissions	15.1	*3.71*	18.7	*5.62*	19.7	*3.75*	*F*_(2,12)_ = 0.32	0.729	0.091
% mounts with intromissions	48.5	*10.3*	40.1	*10.4*	51.7	*4.43*	*F*_(2,12)_ = 0.34	0.716	0.093
Total duration of investigation (s)	32.4	*7.46*	42.7	*19.7*	28	*13.4*	*F*_(1.2,7.3)_ = 0.25	0.678	0.073
% time spent investigating^a^	4.32	*0.85*	4.34	*1.86*	2.82	*1.25*	*F*_(2,12)_ = 0.36	0.708	0.094
% time spent investigating while laser ON^b^	-	-	5.4	*2.62*	7.87	*5.44*	-	-	-
% time spent investigating while laser OFF^c^	-	-	9.58	*2.16*	9.03	*1.79*	-	-	-

†F tests are from one-way, repeated measures ANOVAs comparing data from baseline, timed, and triggered tests; Hunyh-Feldt correction was applied where appropriate; *n* = 7 for each test.

* Tests lasted until an ejaculation was achieved or until 20 min had elapsed; the first 2.5 min was allowed for habituation during which no data were collected.

^a^Defined as (total investigation time/test duration) × 100.

^b^Defined as (investigation time while laser ON/laser ON duration) × 100.

^c^Defined as (investigation time while laser OFF/laser OFF duration) × 100.

Meanwhile, measures of investigation of the female by male subjects in the three tests, including total duration of investigation or percentage of the test spent investigating were not different (Table 3), indicating that in contrast to the effects of laser on the investigation of diluted female urinary odors observed in the odor choice tests, optogenetic activation of AOB M/T cells did not enhance investigation of the female during dyadic interactions. In particular, no differences were observed in the percentage of time spent investigating during the laser-OFF versus laser-ON periods in the triggered tests (*t*_6_ = 0.253, *p* = 0.81, power = 0.06); and in the timed tests the percentage of time spent investigating during the laser-OFF period was actually greater compared with the laser-ON period (*t*_6_ = 2.8, *p* = 0.03, power = 0.65).

## Discussion

In male mice in which blue light-sensitive ChR2 was expressed bilaterally in a relatively small subpopulation of AOB projection neurons, laser stimulation produced odor context-dependent responses. During investigation of diluted EFU, which normally has reduced attractiveness in comparison to undiluted EFU, concurrent laser activation of AOB M/T cells increased investigation, whereas investigation of diluted IMU was decreased when the same AOB laser stimulation was applied. The opposite response to the two odors is notable because the repeated measures design enabled us to examine, in each male subject, the effects of laser-induced activation of the identical population of ChR2-expressing AOB M/T cells. Thus, the same parameters of optogenetic AOB stimulation can augment or reduce males’ investigation of urinary stimuli, suggesting that the AOB input pathway is a dedicated motivational forebrain circuit that modulates hard-wired approach/avoidance responses to pheromones derived from the two sexes.

It is tempting to speculate that laser-induced activation of the AOB inputs to the forebrain accentuated the incentive value of diluted urine to the level of undiluted urine or beyond. Indeed, in the odor choice tests, investigation of diluted EFU was accentuated by optogenetic activation of the AOB, although it did not equal the level of investigation observed with 100% EFU. The results for IMU are more difficult to interpret, as responses of male mice to IMU appear less consistent compared with EFU. For example, Pcdh-Cre/AAV-ChR2 mice in group 1 showed responses to 5% IMU that were no different from responses to 100% IMU (Fig. 4*B*), a response that was replicated using noninfected control mice (C57Bl/6) in group 3 (Fig. 6*B*). To examine this further, we tested a group of Pcdh-Cre^-/-^ male littermates in the olfactory choice box to measure investigation of 100% IMU versus water followed by 5% IMU versus water. Again, there was no difference in the investigation of 100% or 5% IMU relative to water (mean ± SEM for water vs 100% IMU = 8.7 ± 1.5 vs 7.5 ± 1.5; water vs 5% IMU = 6.9 ± 1.7 vs 8.3 ± 1.8). Thus, the laser-induced reduction in investigation of 5% IMU by Pcdh-Cre/AAV-ChR2 males suggests that 5% IMU is aversive when coupled with laser activation, reducing investigation below that exhibited to either 100% IMU or 5% IMU. It is interesting to note that in contrast to the pilot study using Pcdh-Cre^-/-^ male littermates (described above), Pcdh-Cre/AAV-ChR2 males preferred to investigate 5% IMU over water (Fig. 4*B*, trials 4 and 6) during tests given 1 d after 5% IMU had been paired with laser activation of the AOB (trials 3 and 5). One interpretation of this result is that the positive valence seen with dilute IMU presented on its own was a consequence of the recent prior pairing of this stimulus with artificial, optogenetic activation of the AOB.

The aversive effect of pairing optogenetic activation with 5% IMU may be related to the known role of the AOS in territorial scent marking and aggression shown by male mice to male intruders (Clancy et al., 1984; Maruniak et al., 1986). Perhaps laser stimulation coupled with diluted IMU caused male subjects to avoid investigating what they perceived as a urinary stimulus from a more dominant/territorial male. We note further that the effects of laser activation on the response to urinary chemosignals depended on the subject having physical access to the urine, as there was no effect of laser applied concurrently with nose-poking when only urinary volatiles were accessible. This observation strongly implicates the essential role of AOS-dependent processing of nonvolatile pheromonal cues in the behavioral response to odors, both in isolation and in a social context (i.e., mating), as discussed below. These results also show that in the present context the ability of optogenetic AOB stimulation to modify males’ investigation of diluted EFU as well as IMU did not reflect the initial detection and processing of these olfactory stimuli by the MOS, as was previously suggested may occur (Xu et al., 2005; Martel and Baum, 2007, 2009).

Results of the mating study provide evidence that enhancement of AOS signaling during investigation of the female augments males’ mating performance. When the laser was turned on, a higher proportion of mounts resulted in penile intromission, but only when laser stimulation was initiated when males were in nasal contact with, or mounted the estrous female. This suggests that strong AOS input concurrent with mounting improves mating efficiency, or penile hit rate. Conversely, there was no tendency for olfactory investigation of the female to be increased by laser in either the triggered or timed mating tests. An explanation may be that investigation is reduced in the presence of the female and replaced by copulatory behaviors. Nevertheless, our results suggest that strengthening input from the AOS during such behaviors improves performance, perhaps by maintaining males’ level of arousal during mating. Previous studies in male mice where the VNO was surgically removed (Pankevich et al., 2004), the AOB was lesioned (Jakupovic et al., 2008), or in which VNO signaling was impaired by knocking out TRPC2 cation channels that respond to social odors (Leypold et al., 2002; Stowers et al., 2002) showed no disruption in mounting behavior. Taken together, our new findings suggest that AOS signaling facilitates males’ mating performance by augmenting their attraction to female pheromones as well as their sexual arousal, leading to improved mating efficiency.

Previous anatomic studies in several species, including rat (Scalia and Winans, 1975), hamster (Davis et al., 1978; Coolen and Wood, 1998), and mouse (Kang et al., 2011), showed that AOB M/T cells project to the anterior, and to a lesser extent the posterior dorsal subdivisions of the medial amygdala. The medial amygdala, in turn, projects to the medial preoptic area as well as the bed nucleus of the stria terminalis (Kevetter and Winans, 1981), brain regions implicated in the control of male sexual behavior in numerous studies (Hull and Rodriguez-Manzo, 2017). In the present study, we have no specific information about the axonal targets in the medial amgydala of the particular groups of AOB M/T cells that we activated optogenetically. Lesions of either the rostral or caudal subdivisions of the medial amygdala disrupt the preference of male hamsters to investigate female pheromones (Maras and Petrulis, 2006). This outcome would suggest that optogenetic activation of AOB M/T cells targeting either of these subdivisions might have mediated the increased investigation of diluted female urinary odors seen in the present study. An early study in male hamsters (Lehman et al., 1980) found that destructive lesions of the rostral subdivision of the medial amygdala more effectively disrupted mating performance than did lesions placed in the caudal subdivision. Extrapolating to male mice, this latter outcome would imply that the ability of optogenetic activation of AOB M/T neurons to facilitate intromissions may have depended on increased activity in the more rostral segments of the medial amygdala. Additional studies are needed to address these questions.

We cannot rule out the possibility that a new percept was created in the presence of a 5% odor cue by concurrent laser stimulation of ChR2-expressing neurons that are not normally activated by the odor. Similarly, a different set of parameters used to activate AOB neurons could have produced different behavioral responses. We selected a 20-Hz frequency at a pulse duration of 5 ms based on previous research using optogenetic stimulation of cells expressing ChR2 in behaving animals (Gradinaru et al., 2009) as well as the rate of mitral cell firing observed in males as they investigate a female conspecific (Luo et al., 2003).

Several observations strengthen the interpretation that laser stimulation selectively affected the response to pheromonal cues in mice infected with ChR2. First, in the odor choice trials, laser stimulation did not affect investigation of urinary odors when nasal contact was prevented and only volatiles were available. Moreover, there was no effect of optogenetic activation of AOB M/T cells on males’ investigation of diluted food odor, whether or not physical access to the stimulus was permitted. There is currently no evidence that food or any other nonsocial odors are processed by the AOS, although one report (Sam et al., 2001) showed that isolated VNO sensory neurons can respond to various odorants. Nevertheless, food (cookie) odor was clearly attractive to mice, so laser stimulation did not strengthen the saliency of all attractive odors.

In a control group of uninfected C57Bl/6 males, laser stimulation had no effect on investigation of urinary odors even when nasal access was permitted, indicating that laser alone did not produce behavioral effects. Although we did not examine mice infected with a null virus, there is also no indication that viral infection per se affected either investigation of odors in the odor choice tests or sexual behavior during the mating tests. In the absence of laser stimulation, male mice infected with ChR2 virus displayed the usual attraction for female urinary odors, and as exemplified in the baseline mating tests, exhibited typical levels of mounting and intromissions with female subjects, and in some tests, achieved an ejaculation.

There was also no effect of laser activation on the investigation of a null stimulus (water) indicating that laser stimulation alone was neither aversive nor rewarding. This indicates that signals created by laser-induced firing of ChR2-expressing neurons require a nonvolatile odor context for such signals to be salient. From the electrophysiological recordings of anesthetized mice that received laser stimulation, we saw that spiking occurred in the medial amygdala even without odors present, suggesting that medial amygdala activation alone is not sufficient to alter investigation of an irrelevant stimulus. The absence of any behavioral effect of optogenetic AOB activation in the absence of a pheromonal odor context contrasts with results of previous experiments showing that optogenetic activation of dopaminergic neurons in the ventral tegmental area (Tsai et al., 2009) as well as serotonergic neurons in the dorsal raphe nucleus (Li et al., 2014) caused mice to investigate stimulation-linked spatial areas.

Spread of the ChR2-carrying virus to a few adjacent dorsal MOB M/T cells was seen in most subjects, so we have not completely ruled out the possibility that optogenetic-induced activation of these cells could have contributed to the behavioral effects documented in this study. There are two reasons, however, why this is unlikely. First, the optical fiber that delivered laser stimulation was implanted along the midline between the left and right AOBs. As laser intensity dissipates through tissue (Yizhar et al., 2011), optical signals reaching MOB M/T cells would be weakened. Although it remains possible that this signal was nevertheless sufficient to activate MOB cells, an important observation was that there was no effect of laser stimulation on group 2 subjects’ preferences for either diluted EFU or IMU, where access to only volatiles was permitted. Thus, even if the MOB was activated by laser in these subjects, there was no effect on investigatory behavior when only volatile chemosignals, which would have been detected and processed via the main as opposed to the AOS, were present.

In conclusion, these results extend previous lesion and genetic disruption studies suggesting that the VNO-AOB inputs to the forebrain motivate male mice to seek out opposite-sex and avoid same-sex pheromones. Moreover, we demonstrate that activation of this circuitry can augment sexual arousal, leading to an increased probability of penile intromission and improved mating performance.

## Synthesis

Reviewing Editor: Cheryl Sisk, Michigan State University Decisions are customarily a result of the Reviewing Editor and the peer reviewers coming together and discussing their recommendations until a consensus is reached. When revisions are invited, a fact-based synthesis statement explaining their decision and outlining what is needed to prepare a revision will be listed below. The following reviewer(s) agreed to reveal their identity: Daniel Wesson.

Both reviews commented that the findings are interesting and novel in suggesting that AOB activity during copulation may be important for ongoing sexual behavior. Both also noted that the experiments are well executed and the manuscript clearly written. The reviewers have several suggestions for improving the manuscript and in some cases ask for additional data or information that should be readily available (i.e., no new experiments or data collection should be required). Please respond to these comments and make appropriate revisions to your manuscript. I will evaluate your responses and make an editorial decision upon receipt of your revised manuscript.

1. In the current description of the “triggered” optogenetic stimulation tests there are behaviorally distinct contexts that could result in triggered stimulation. It would be helpful for the authors to clarify the operational definition for investigation/stimulation trigger. Specifically, what duration of contact was required to be considered investigation? Additionally, if the animals were in contact at the offset of the 2.5min period post stimulation and remained in contact after the 2.5min mark was this considered a new bout of investigation and would this trigger a new onset of optogenetic stimulation?

2. Might the authors be able to provide a very rough quantification or at least an estimate of the number (or even range) of off-target transduced neurons in the MOB? They report transducing </=6% of all AOB MT neurons. Their description of the MOB labeling suggests transduction was far lower in those neurons. Could the authors give just a small amount of additional detail here? Note I am not requesting new data be collected since the authors already commented upon the extent of this transduction. Simply more elaboration would be helpful.

3. It would be helpful for the authors to mention how closely this light stimulation paradigm might shape the firing rate of these M/T neurons in manners similar (or not) to their endogenous odor-evoked firing rates.

4. Some minor details would be helpful for replication purposes. 1) Exact stereotaxic coordinates for the optical fiber implantation and 2) the rate the micropipette was withdrawn from the brain post injection.

5. In several locations there is data not presented (“data not shown”) but some of it is quite important and should be easily presented in tables. Notably, the investigation times and other copulatory parameters in the sex tests need to be shown.

6. There is a minor concern with the lack of null virus controls during the experiments as viral vector introduction to the AOB may have behavioral effects on its own. However, this concern is mitigated by the experimental design in which the Timed stimulation had no effect during copulatory testing and the limited set of tests in wild-type animals. The authors might want to comment on this in the manuscript.

7. There does appear to be some sort of rebound effect or enhancement of dilute IMU investigation after IMU+light pairing. Please discuss as this seems to suggest a change in valence/interest toward these odors (they now appear to be investigated greater than water).

8. The authors conclude their data result in “reproductive success”. This isn't supported and isn't necessary either for the paper to stand on its own. The increase probability of a consummatory sexual behavior, itself, without showing increased fertility rate, doesn't directly link these results with “reproductive success”.

9. In the Discussion, it is not clear why the extensive discussion of the Haga-Yamanaka et al 2014 paper is warranted. In addition, is it possible that increased activation of the M/T cells increases detection of chemosignals rather than changing valence?

10. In the discussion, could the authors expand upon on the potential role of the medial amygdala as it relates to the increased frequency of mounting with intromission resulting from triggered AOB optogenetic stimulation? This could potentially strengthen the support for the role of the AOB in sociosexual behavior. For instance, do we know what neurons AOB M/T cells synapse upon? Where do those neurons project which may influence these sexual behaviors?
